# Account of a Case of Choleroid Affection, Produced by the Poison of Muscles

**Published:** 1834-10-01

**Authors:** Theophilus Thompson

**Affiliations:** Physician to the Northern Dispensary.


*Account of a Case of Choleroid Affection, produced by the Poison of Muscles.* Communicated to the Harveian Society, by THEOPHILUS THOMPSON, M.D., Physician to the Northern Dispensary.

WILLIAM HOOPER, a tall, muscular man, aged fifty, came to me for advice, to the Northern Dispensary, October 7th, 1833. He was evidently labouring under great debility: streams of perspiration were running down his forehead; his breathing was short and interrupted; his countenance peculiarly pallid and anxious, and his lips were of a deep hue; the hands were extremely cold; the pulse, just sufficiently perceptible to be counted, was 100; the tongue much furred; and the epigastrium very tender on pressure. He complained of pain and a sensation of heat in that situation, which had lasted all day. Since the previous morning he had vomited all his food. Much diarrhœa yesterday; today less considerable. The respiratory sounds were natural, and the urine passed freely. No cramp, itching, or numbness. I questioned the man closely respecting his diet during the week, but he did not mention anything likely to produce the symptoms.

Six leeches to be applied to the epigastrium. To have two grains of calomel and half a grain of opium every two hours. Chalk mixture, with a few drops of aromatic spirit of ammonia, and tincture of henbane, after every loose evacuation. To drink freely of barley-water holding gum arabic in solution.

It was agreed that a messenger should bring me an account of the patient in the evening.

October 8th. Having heard no account of the man on the previous night, I called at the house, and found him in a state very similar to that in which he appeared on the day before. The perspiration was still streaming down his face and chest; the extremities were cold, and the pulse 100 and regular, but scarcely perceptible, from its weakness; the action of the heart not strong, but could be distinctly heard over the lower part of the right side of the chest. The respiratory murmur was heard over the greater part of the chest. He evinced great anxiety and restlessness, frequently rising, and commencing a sentence with much animation, but lying down again before it was completed. The tenderness of the epigastrium was considerable, and the tongue was covered with a brown fur. There had been only one evacuation, which was reported dark, but had been thrown away. The leeches, before they were half full, had fallen off dead: the blood was extremely black. Vomiting still frequent.—He was directed to take tartrate of soda, with tincture of henbane, and to continue the pills of calomel and opium.

Although the case presented a hopeless aspect, yet, as the inflammatory symptoms were distinct, and the pulse remained unchanged, it appeared to me proper to try the experiment of bleeding, premising the use of the hot-air bath. The patient was to be kept in the erect posture during the operation, the finger to be kept on the pulse, and the blood at once stopped if the pulse should not rise. A small quantity was taken from the right arm; it trickled down thick and black, but soon stopped. The pulse having risen a little, a vein was opened in the left arm; an ounce and a half of blood was withdrawn in a slender stream, without any decided effect on the pulse, when it suddenly ceased to flow, and the patient fell back in a state of syncope, from which he never recovered.

*Sectio Cadaveris.* The body was inspected forty-four hours after death, with the valuable assistance of my excellent colleague, Dr. Stroud.

External surface: The body was well made, large, muscular, and moderately fat. The integuments of the sides and back had a livid aspect. This livid appearance was most observable in the arms, particularly in the course of the veins, and near the punctures made in venesection, which were still slightly bleeding, and surrounded with ecchymosis. The superficial veins of the neck, chest, and arms, were much distended. Percussion of the chest gave a dull sound. There was no shrinking of the extremities: a silver ring on one of the fingers could not be removed, either during life, or after death.

Thorax: The pleuræ were pale. The right pleura, at its upper part, presented many firm, short adhesions. The left pleural sac contained two quarts, and the right one quart, of transparent serous effusion. The lungs were collapsed and soft. The right lung adhered rather firmly to the diaphragm, and some vesicles at the edge of the lower lobe were emphysematous. The pericardium contained nearly a pint of citron-coloured serum.

The heart was of the natural size. The right auricle full of dark blood, by which this auricle, the right ventricle, and the pulmonary artery, were deeply tinged.

Abdomen: The peritoneum was in several places reddened, and in some parts, where it covered the ascending colon, distinctly ecchymosed. The colon was dilated to nearly twice its natural size; its transverse arch, bending down at an abrupt angle, in the middle descended below the umbilicus, then rising to the left hypochondrium, pursued its usual course. The sigmoid flexure was contracted, but healthy. The mucous membrane of the stomach was very vascular, and, with the exception of a small part near the lesser curvature, presented the appearance of a bright red patch tinged with grey. This appearance, when closely examined, was found to arise from hemorrhagic depositions of a capillary and punctiform character. There was a hemorrhagic spot, the size of a pea, near the pyloric extremity. Many venous trunks, of considerable size, were seen ramifying along the great and small curvatures.

The mucous membrane of the colon exhibited a number of patches, of a round, oval or irregular form, and mottled aspect, from one fourth of an inch to two inches in diameter; some of a nearly uniform livid red, others of a deep red, nearly approaching to black. These colours seemed to be produced partly by venous congestion, but chiefly by sanguineous effusion. Over the brighter patches the mucous membrane was little changed; over those of deeper tint, it was in a state of considerable vascularity and congestion. Over one of the largest patches a circular portion of the mucous membrane, half an inch in diameter, was soft, and of a dirty yellowish grey, presenting the character of a slough. Another portion near this, of similar form and size, appeared in a less advanced stage of sphacelus, of a deep green colour, and traversed by minute veins gorged with blood. At this part, and wherever the hemorrhage was considerable, the mucous membrane was softened; in all other parts its thickness and consistence were natural, and its colour was for the most part reddish yellow, with a tinge of grey and blueish green.* It could be made to slide easily over the patches. The small intestines presented some congestion, chiefly ramiform, and a few small patches, of a bright red colour. The follicular structure was nowhere affected. The intestines contained a considerable quantity of feculent matter.

The liver was of moderate size: much dark blood followed incisions made into its substance. The gallbladder was full of dark bile, which was squeezed with difficulty through the ducts. The urinary bladder was empty.

The appearances on the inspection producing strong impressions that some unwholesome diet had been taken, careful inquiries were made upon the subject, and it was for the first time discovered that, on the evening of October 4th, four days before his death, this poor man had eaten about a pint of muscles, which were plump and fresh; they were boiled, and taken with pepper. The widow afterwards gave the following particulars of the progress of his attack: After taking the muscles he had slept pretty well, and the next day went to work, but returned at one o'clock to dinner, complaining of weakness and a peculiar feeling in the head, which he supported on his hand, and went to sleep. His wife awoke him, but he again fell asleep. After repeatedly dozing, and being repeatedly aroused, he took a little bread and cheese and beer, and went back to work. In the evening he took a little coffee. At two o'clock on Sunday morning, October 6th, he awoke with diarrhœa. In the afternoon sickness came on, and his hands became cold. He took a little apple-pudding for dinner, and afterwards some broth, but immediately after complained of violent pain in the loins. His wife rubbed him with spirits of turpentine, from which he experienced relief. On Monday morning, October 7th, he complained that exactly the same pain he had felt in the loins had come to his stomach.

On Sunday his bowels were purged six times, on Monday four times. He experienced no relief to the vomiting from its commencement, on Sunday, till a short time before his death. There was no itching nor eruption of the skin. He passed no urine during the last twenty-four hours.

He had tended a mill connected with a brush manufactory, and was much exposed to dust, which had occasionally produced fits of coughing; but this had been his only complaint, excepting an attack of pain in the stomach, (six weeks before

* Dr. Carswell has executed with his accustomed accuracy a beautiful drawing of the stomach, and of the part of the colon most affected; and, to ensure fidelity, I have to a great extent adopted his expressions, in the above description of the appearances of those parts.

his death,) which was immediately relieved by a powder obtained from a druggist.

Remarks. This case may be considered as an interesting example of the effects which may be produced upon the heart and capillary system, by a shock communicated to the stomach and intestines, and diffused, through the medium of the nervous influences, over various parts of the system.

It may serve to illustrate several of the more remarkable phenomena presented by malignant cholera, to which disease the case under review presents some striking analogies. There was the same failure of the circulation, both general and capillary, with a deterioration of the quality of the blood. The extreme coldness; the relaxed state of the capillaries of the skin, causing streams of perspiration to run down various parts of the body; and, ultimately, the suppression of urine, present additional points of resemblance. The failure of the circulation, and the deterioration of the blood, must in this case be referred rather to the influence of some septic cause, than to the diarrhœa and vomiting, which were insufficient to produce such effects. The evidence for the existence of some septic influence seems confirmed, when we consider that, from the time the patient's extremities became cold, there was no decided effort at reaction: and, when reaction fails to follow congestion in the large veins, it seems reasonable to conclude that some sedative cause prevents the heart from being excited by an unusual quantity of its natural stimulus.*

It is only by allowing the existence of a similar cause, that we can explain many of the phenomena of malignant cholera. The very first symptoms are frequently enfeeblement of the nervous power. The excitement of inflammatory action sometimes relieves the nervous depression; but, when reaction is insufficient for that end, the sedative cause prolongs its influence, and introduces a new train of symptoms. Hence the occurrence of typhoid symptoms, after recovery from the collapse of cholera.

The effect of this influence on the organic system of nerves appears analogous to that which is apt to arise from severe mechanical injury.† When dangerous injury of this nature does not immediately destroy life, the subsequent fever generally assumes a typhoid character, and any accompanying inflammation tends rapidly to gangrene.

In the case of Hooper, the existence of such a cause is not

* Vide Alison's Pathology, *passim*.

† Philip on the Laws of Organic Life. M. Hall, Researches on the Circulation.

a matter of speculation, and the post-mortem effects are of a nature not otherwise to be explained. The accuracy of our reasoning being in this case demonstrated, a strong presumption arises in favour of a conclusion, to which the same train of argument conducts us on the subject of cholera. What the specific cause may be in that disease, and to what extent analogous in its operations to the cause of the present case, are legitimate objects of inquiry. The effusion into the pleural sacs was an unexpected event; it must have occurred very rapidly. Some may attribute it to obstructed circulation, but we know that concussion does occasionally produce dropsy, and perhaps in this case the effusion might be the consequence of a similar nervous impression made on the capillary vessels. When the pleura is at all changed in cases of cholera, it is for the most part drier than natural: but may not serous diarrhœa counteract any tendency to effusion from serous membranes?

The morbid anatomy of cholera has hitherto failed to afford a satisfactory insight into the nature of diseased actions which constitute it, and has proved instructive rather in the way of negative than positive information. But we are not without descriptions of appearances in the intestines of those who have died of cholera similar to those in the case before us. Dr. Jackson, of Philadelphia, in his admirable narrative, mentions one case, where the collapse was rapid, in which "red patches were scattered through the internal surface of the stomach; the mucous membrane of the colon at first glance might be supposed to be sphacelated, but, on examination, the consistence of the membrane was found to be perfect, and the black hue proved to arise from blood effused into the submucous cellular tissue, or rather the derma of the dermoid tissue." In another case, "the mucous membrane of the cardiac extremity of the stomach was of a deep red colour; in the great curvature were patches of intense red, caused, by infiltration of blood into the tissue; stellated patches were observed at the pyloric extremity."*

Similar appearances are detailed by Annesley, Keir, Brière and Legallois, Searle, &c.

So far as we observed in Hooper's case, the follicular structure was not diseased, but in almost all the cases of cholera in which the intestines were much affected, the glands of Peyer and Brunner suffered.

It may be proper to consider to what extent the symptoms in the case before us corresponded with the ordinary symptoms of fish-poisoning.

* American Journ. of Med. Sciences, May 1833.

The most remarkable effects produced by poisonous fish are, general uneasiness, precordial anxiety, difficult respiration, frequent, small pulse, nausea, pain at the epigastrium, swelling of the face, then of the body, which is intensely red or covered with white petechiæ, more or less elevated, preceded by itching; cold sweats; sometimes coryza and asthma.

The great variety of parts affected in different individuals from the influence of the same cause, is remarkable. These differences appear to arise partly from the varying strength of the poison, partly from predisposition. The absence of numbness, prickly sensations, or urticaria, in Hooper's case, although remarkable, can scarcely be regarded as opposing the view I have offered. The occurrence of urticaria is probably rather favourable than necessary. It is a kind of reaction that evinces vital energy, and it may relieve the nervous oppression. The appearance of a scarlet rash in cholera is generally considered favourable; and in a case of poisoning from muscles, which occurred at Paris, a sense of imminent suffocation immediately followed the suppression of the eruption.

In most of the cases of poisoning with muscles which occurred at Leith, in the year 1827, there was no immediate local action on the stomach, but it appeared (to use the words of Dr. Combe,) that "the virus was first absorbed, and exerted its influence on the nervous energy." The first symptoms were those of debility, then followed numbness, a prickly sensation in the hands, cold surface, vertigo, sometimes, but not constantly, nausea and cardialgia.*

In all severe cases the action of the heart was feeble. In some the secretion of urine was suppressed. Those that died went off as in a gentle sleep, and spoke rationally to the last. The same peculiarity was observed in Vancouver's expedition, in a sailor, who died six hours after taking muscles. Hooper died calmly, as though from syncope, or increasing weakness, retaining his intellectual faculties to the last unimpaired; a peculiarity which Orfila and others specify as a distinctive characteristic effect of septic poison.†

Our medical records offer but few accounts of post-mortem inspections of those who have died from fish-poisoning; but those which are detailed accord with the appearances in Hooper's case. Foderé mentions,‡ as a general appearance

* Edinburgh Med. and Surg. Journal, vol. xxix.

† Toxicol. Generale, tom. ii. p. 449.—The effects of septic poisons have been summed up as follows: "Weakness, syncope, cold sweats, universal paleness, hæmorrhagy, extinction of the force of the heart, prompt death, with clear intellect. After death, flexibility and speedy putrefaction."

‡ Médecine Légale, vol. iv.

from poisoning with muscles, slight inflammation of the stomach. In one of the cases examined by Dr. Combe, several red patches were found in the small intestines. In the other the abdomen was tumid, the ileum presented some patches of effusion of a dark red colour, and the colon was constricted.

The limits of this paper will not allow an extended discussion of the question to which this case might lead, on what does the deleterious influence of poisonous fish depend? The opinions of Mohring, that it arises from the Stella marina; of Kerner, that it depends on margaric or oleic acid; and of others, that it is occasioned by a peculiar empyreumatic oil, appear equally incapable of proof; but the statement of Lamaroux, that muscles are apt to become poisonous when exposed alternately to the sea and the air, is favoured by some of the most striking instances on record.

Putrefaction does not destroy, but perhaps rather conduces to the development of the poisonous principle, and, as fish decay more speedily than warm-blooded animals, and diseased fish more speedily than others, this cause must be frequently in operation. In the fatal cases related by Dr. Burrows, the muscles were tainted. Foster, Quarrier, Thomas Clark, Kœmpfer, and others, agree that the same fish which is innocuous when fresh, may become poisonous when stale.* The case before us, however, is one of many which prove putrefaction not to be essential, as the muscles were plump and fresh.

This case offers no support to the opinion of Dr. Stone, Edwards, and others, that the effects depend on idiosyncrasy. Hooper's wife had on several occasions suffered from partaking freely of muscles, and in this instance probably owed her immunity to the small quantity she took.

Rondeau asserted that the deleterious effects of poisonous muscles might be obviated by boiling them with vinegar and water, and cayenne pepper. Salt has been long observed to be an antidote to the Clupea thryssa, Barra cuda, and other fish; but if incorporated with the fish, as in the case of red herrings, it seems to be inefficacious.

Of all the substances that have been recommended, sugar, and the juice of the sweet potato (*Convolvulus Battatas*), appear best entitled to the name of antidotes.†

* Edinburgh Med. and Surg. Journal, vol. iv. (Chisholm.)—There seems to be considerable evidence for the opinion that poisonous fish are the subjects of disease, changing their secretions, and producing a peculiar virus, not appreciable by our present modes of investigation. It is a remarkable fact, that at the same season the yellow-billed sprat, caught off Porto Rico, is innocuous; and the same species, taken at the neighbouring island of Eustathia, deleterious in the extreme.

† Edinburgh Philosophical Journal, vol. i. (Dr. Ferguson.)

It is obvious that the first indication in cases of fish-poisoning, is to relieve the stomach of the noxious ingesta, by the aid of emetics of sulphate of zinc or copper. When this has been accomplished, without material relief, we have to determine whether the symptoms evince a predominance of the sedative influence, or of the inflammatory state produced by reaction. Here the treatment of fish-poisoning, and that of some cases of cholera, with early exhaustion, appear to illustrate each other; and we are perhaps authorised in saying, that the most alarming symptom in each is depression of the heart's action, and that the use of stimulants is in the first instance often essential.

Ether has been found very successful by Dulong, Charlet, Montègre, Demangeon,* and Dr. Combe. Capsicum, and the oxymuriate of potash, have also been recommended on very high authority. Had Hooper applied for advice at the time the drowsiness first appeared, it is probable that ether, ammonia, or some other diffusible stimulant, might have saved his life. The case might then have become one of simple inflammation; but it does not appear that the tendency to enteritis in cholera (and analogous affections) contraindicates the use of stimulants at the onset of the disease.

It is an allowable inquiry, whether the abstraction of blood might not, in some cases, contribute to produce reaction. When inflammatory symptoms set in, and become predominant, we may reasonably expect to arrest the disease by the aid of the lancet, and by the general adoption of antiphlogistic measures.

* Gazette de Santé, Mars et Octobre, 1812, and Mars, 1813.

